# Parenting and adolescent anxiety within families: a biweekly longitudinal study

**DOI:** 10.1111/jcpp.14161

**Published:** 2025-03-19

**Authors:** Lucija Šutić, Ezgi Yıldız, F. Cemre Yavuz Şala, Aylin Duzen, Loes Keijsers, Savannah Boele

**Affiliations:** ^1^ Faculty of Educational and Rehabilitation Sciences University of Zagreb Zagreb Croatia; ^2^ Department of Psychology Boğaziçi University Istanbul Turkey; ^3^ Department of Educational Psychology Ankara University Ankara Turkey; ^4^ Department of Psychology University of Bologna Bologna Italy; ^5^ Department of Psychology, Education & Child Studies (DPECS) Erasmus University Rotterdam Rotterdam The Netherlands

**Keywords:** Adolescence, generalized anxiety symptoms, intrusiveness, autonomy support, within‐family level

## Abstract

**Background:**

Anxiety symptoms among adolescents have been increasing globally. The present study aimed to better understand the role of parenting, which is believed to act as both a risk and protective factor for anxiety while also being impacted by adolescent anxiety. Specifically, this preregistered study examined the bidirectional associations between parental autonomy support, intrusiveness, and symptoms of generalized anxiety in adolescents.

**Methods:**

We used meso‐longitudinal data of Dutch adolescents (*N* = 256, *M*_age_ = 14.4, age range = 12–17, 71.5% female, *t*_mean_ = 17.7) and their parents (*N* = 176, *M*_age_ = 46.8, 82% female, *t*_mean_ = 22). They reported biweekly on parental intrusiveness and autonomy support and on adolescent generalized anxiety symptoms. Dynamic structural equation modeling (DSEM) was used to examine the associations at the between‐ and within‐family levels.

**Results:**

The between‐family level associations indicated that adolescents from families with lower levels of parental autonomy support and higher levels of parental intrusiveness exhibited higher levels of generalized anxiety symptoms. Within families, during weeks when parents were less autonomy supportive or more intrusive, adolescents also experienced more generalized anxiety symptoms. Regarding the bidirectional time‐lagged effects, adolescent‐driven, but not parent‐driven, effects emerged. Specifically, when adolescents experienced more generalized anxiety symptoms than usual, their parents were less autonomy supportive and more intrusive 2 weeks later.

**Conclusions:**

Although further research is needed, these findings underscore the negative impact of adolescents' mental health issues on parenting. To prevent the further escalation of family problems, it seems vital to promote positive and adaptive parent–child interactions when adolescents face mental health issues.

## Introduction

Anxiety symptoms are increasing globally and are among the most common mental health problems among adolescents (WHO, 2022). Symptoms of *generalized* anxiety include uncontrollable and excessive worries about a wide range of domains, including social situations, performance, natural events, perfectionism, health, and family (Ellis & Hudson, 2010). These worries are often accompanied by physical symptoms such as restlessness, chills, or hot flushes (McLellan & Hudson, 2017). Given that generalized anxiety can be triggered by many everyday situations and that generalized anxiety disorder is one of the most prevalent anxiety disorders in adolescents (Steinsbekk, Ranum, & Wichstrøm, 2022), understanding the causes and consequences of adolescents' generalized anxiety symptoms is crucial.

Parenting is widely recognized as a key factor in the prevention of adolescent anxiety (Biswas et al., 2020; Yap et al., 2019; Yap, Pilkington, Ryan, & Jorm, 2014). However, few empirical studies have examined how parenting practices contribute to changes in adolescents' generalized anxiety symptoms over time within families (Boele, Denissen, Moopen, & Keijsers, 2020). To address this gap, we examined whether parenting practices, specifically autonomy support and intrusiveness, predict biweekly fluctuations in adolescents' generalized anxiety symptoms and whether this biweekly relationship is bidirectional.

### Linking parental autonomy support and intrusiveness to adolescent anxiety

Parenting is a modifiable etiological factor in adolescent anxiety and thus may serve as a target for preventive interventions (Yap et al., 2019). Parents can reduce or induce anxiety in their children through positive and negative parenting practices (Murray, Creswell, & Cooper, 2009; Yap et al., 2014). The Self‐Determination Theory (SDT) provides a useful framework for understanding how parenting affects adolescent anxiety. The SDT posits that three psychological needs—autonomy, competence, and relatedness—are essential for healthy functioning (Ryan & Deci, 2017). Parents can support these universal needs and, in turn, adolescents' well‐being through various practices, including autonomy support, involvement, and structure (Ryan & Deci, 2017; Soenens, Vansteenkiste, & Beyers, 2019). Conversely, intrusive, neglectful, or harsh parenting can undermine these needs and negatively impact adolescent well‐being. The present study examined whether parental autonomy support improves adolescent psychological well‐being (i.e. fewer symptoms of generalized anxiety) and whether parental intrusiveness undermines adolescent psychological well‐being (i.e. more symptoms of generalized anxiety).

Parental autonomy support involves fostering adolescents' growing need for volitional functioning. Parents can foster this by allowing them to make their own choices and form their own opinions (Soenens et al., 2019; Soenens, Deci, & Vansteenkiste, 2017). Moreover, parents who support their adolescent's autonomy minimize control, show empathy for their needs and interests, and encourage exploration of their identity (Grolnick, 2003; Ryan, Deci, & Grolnick, 1995). When children's need for autonomy is satisfied, they experience a sense of volition, ownership of their own behavior, and confidence in their ability to cope with challenging situations (Soenens et al., 2017, 2019). Autonomy‐supportive parenting thus helps children develop age‐appropriate independence from their parents and a sense of competence, control, and mastery (Soenens et al., 2017; Wood, McLeod, Sigman, Hwang, & Chu, 2003). A sense of competence and control over one's behavior is crucial for anxiety regulation, such as worry (Ellis & Hudson, 2010), when faced with new or challenging situations (Weems & Silverman, 2006). Recent research has indeed suggested that autonomy‐supportive parenting plays a role in reducing everyday negative affect (Bülow et al., 2022), a core characteristic of anxiety and depressive symptoms (Dozois, Seeds, & Collins, 2009). Hence, autonomy‐supportive parenting is expected to reduce the symptoms of generalized anxiety in adolescents by enhancing their feelings of competence and control.

Parental intrusiveness stands in contrast to parental autonomy support, although low autonomy support does not necessarily equate to being intrusive (Silk, Morris, Kanaya, & Steinberg, 2003; Soenens et al., 2017). When parents are intrusive, they attempt to control their child (Barber, 1996), which actively thwarts the child's universal need for autonomy (Soenens et al., 2017). Intrusive parents interfere with areas within a child's personal domain, which do not necessarily require parental intervention (Choe et al., 2023; Soenens et al., 2017). This can range from autocratic parental decision‐making to the excessive regulation of children's routines and overprotection (Wood et al., 2003). Hence, intrusive parenting encourages dependence on parents (Wood, 2006), limiting opportunities to learn to deal with difficult or new situations and signals to the child that they are incapable of coping. This may hinder adolescents' development of their competence and mastery. As a result, adolescents may perceive future challenges as beyond their control, increasing their worry (Weems & Silverman, 2006; Wood et al., 2003), which is linked to anxiety and depression (Dozois et al., 2009). Indeed, research indicates that intrusive parenting is related to lower levels of child competence (e.g. Affrunti & Ginsburg, 2012) and higher levels of separation anxiety (e.g. Wood, 2006). Thus, intrusive parenting is expected to heighten symptoms of generalized anxiety in adolescents by undermining their feelings of competence and control.

### Adolescent anxiety contributing to maladaptive parenting

The association between parenting and adolescent anxiety is likely reciprocal in nature (Bell, 1968; Soenens & Vansteenkiste, 2020). There are several theoretical ideas as to why adolescent anxiety may elicit maladaptive parenting, such as less autonomy‐supportive and more intrusive parenting. Interpersonal views on internalizing problems (Joiner & Coyne, 1999) suggest that internalizing symptoms (e.g. anxiety symptoms) may elicit less supportive behavior from significant others, including parents (Branje, Hale, Frijns, & Meeus, 2010), due to aversive interpersonal behaviors. Adolescents with generalized anxiety symptoms often engage in excessive reassurance seeking to cope with uncontrollable worries (In‐Albon, Wahlund, & Perrin, 2020). This behavior in the parent–child relationship can place an emotional burden on parents, making it difficult for them to respond in autonomy‐supportive ways and more likely to respond in an intrusive and controlling manner (Soenens & Vansteenkiste, 2023). Relatedly, adolescents' anxiety symptoms can act as a stressor for parents, potentially negatively impacting their parenting (Belsky, 1984). Empirical research indeed indicates that adolescent social anxiety (Nelemans et al., 2020) and parental stress (Aunola, Viljaranta, & Tolvanen, 2017; Van Der Kaap‐Deeder et al., 2019) are followed by less autonomy‐supportive and more psychologically controlling (intrusive) parenting. Furthermore, child anxiety may prompt intrusive parenting behaviors, as parents attempt to help their child, although in an overprotective and intrusive manner (Segrin, Woszidlo, Givertz, & Montgomery, 2013; Zhang & Ji, 2024). Thus, when adolescents experience more generalized anxiety, their parents are expected to respond with less autonomy‐supportive and more intrusive parenting.

### Empirical research on within‐family processes over time

There are limited empirical studies on within‐family parenting processes, as most involve between‐family group‐aggregated research designs (e.g. Boele et al., 2020; Yap et al., 2014). Between‐family level findings indicate that adolescents who experience less parental autonomy support and more intrusiveness generally experience more anxiety symptoms (Yap et al., 2014; Zhang & Ji, 2024). As within‐family overtime processes cannot be inferred from between‐family designs (Hamaker, 2012; Keijsers, 2016), the underlying transactional parenting processes within families thus remain unclear (Boele et al., 2020).

To date, empirical within‐family studies on parenting and adolescent anxiety remain limited (review: Boele et al., 2020). One of the few related studies used dyadic daily diaries to assess adolescent emotional problems (including anxiety) and parental psychological control—emotionally manipulative behaviors undermining the child's autonomy (Barber, 1996; Soenens et al., 2017). Positive effects were found between psychological control and emotional problems, though the direction of effects depended on whether the parent or adolescent was the informant (Xu & Zheng, 2022). Another annual study indicated effects from adolescent *social* anxiety symptoms to parental psychological control and autonomy support (Nelemans et al., 2020). While social anxiety primarily negatively impacts social functioning, *generalized* anxiety can have an impact on various aspects of life. Therefore, there is also a need to examine how parenting is linked to adolescents' generalized anxiety symptoms over time.

Moreover, earlier within‐family studies focused on either micro‐ (e.g. hours, days; Bülow et al., 2022; Xu & Zheng, 2022) or macrotimescale processes (e.g. years; Nelemans et al., 2020). Nonetheless, meso‐timescales, such as a biweekly timescale, are of theoretical interest (Wood et al., 2003). Both bio(psychosocial) ecological models (Bronfenbrenner, 2005; Sameroff, 2010) and the dynamic systems perspective (Granic, Dishion, & Hollenstein, 2008; Smith & Thelen, 2003) consider recurrent microtimescale interactions as drivers of developmental change (see also Keijsers, Boele, & Bülow, 2022). Hence, to ‘proximal processes’ (i.e. “enduring forms of interaction in the immediate environment”; Bronfenbrenner, 2005, p. 6) between parenting and adolescent anxiety symptoms, a biweekly meso‐timescale might be suitable (Wood et al., 2003).

Indeed, studies have indicated that within‐family parenting effects can be observed on meso‐timescales. A multiple‐timescale study found effects at biweekly and three‐monthly timescales, but not at the commonly studied daily and annual timescales (Boele, Nelemans, et al., 2023). Additionally, research using the same dataset as the current study suggests that the effects of other practices (i.e. warmth and psychological control) on adolescent outcomes (i.e. depressive and anxiety symptoms, and self‐esteem) emerge on a biweekly timescale (Boele, Bülow, de Haan, Denissen, & Keijsers, 2024). Hence, to gain more knowledge about the timing of the processes (Wood et al., 2003), we tested the within‐family associations between parental autonomy support and intrusiveness and adolescents' generalized anxiety symptoms on a novel biweekly timescale. Such biweekly insights can be valuable for prevention programs by identifying the optimal timescales at which changes might occur within families.

### The current study

This current study had three aims. First, to replicate prior work (for a review, see Yap et al., 2014), we examined whether parenting practices are related to adolescents' generalized anxiety symptoms at the between‐family level (Aim 1). We hypothesized that parental intrusiveness would correlate with higher levels of generalized anxiety symptoms (H1a) and parental autonomy support (H1b) with lower levels of generalized anxiety symptoms at the between‐family level. Second, we examined whether the parenting practices and adolescent generalized anxiety symptoms are correlated at the within‐family level (i.e. overtime cofluctuations within the same family). We hypothesized that increases in parental intrusiveness (H2a) and decreases in autonomy support (H2b) would be associated with same‐time increases in generalized anxiety symptoms. Third, we examined the bidirectional time‐lagged effects between the parenting practices and adolescent generalized anxiety symptoms at the within‐family level. We hypothesized that increases in parental intrusiveness (H3.1a) and decreases in parental autonomy support (H3.1b) would predict increases in symptoms of generalized anxiety 2 weeks later. Conversely, we hypothesized that increases in generalized anxiety symptoms would predict increases in parental intrusiveness (H3.2a) and decreases in parental autonomy support (H3.2b) 2 weeks later. To test these preregistered hypotheses (https://osf.io/tfjue/), data were used from families who were followed for a full year, resulting in 26 biweekly assessments by multiple informants (adolescent and parent).

## Methods

### Study design and procedure

In the “One size does not fit all” study (for more information, see http://osf.io/e2jzk), 176 parents and 259 adolescents reported on their well‐being and parenting every other week for a full year (*t* = 26 assessments). Adolescents (aged 12–17 years) and their parents were recruited at Dutch high schools through class visits, parent‐evenings, and the school newsletter. Adolescents and parents provided active informed consent. Parents of adolescents under the age of 16 also provided active consent for the participation of their child. The Ethics Committee of the Faculty of Social and Behavioral Sciences of Tilburg University (Nr. EC‐2019.65t) approved the study.

### Sample

Of the 259 participating adolescents (*M*_age_ = 14.39, *SD*_age_ = 1.585, age range = 12–17 years, 71.5% female, 28.5% male), 256 adolescents provided data for our variables of interest. Most of these adolescents were born in the Netherlands (97%). Regarding schooling, 15% attended (pre)vocational secondary school, 32% attended higher general education, and 49% attended preuniversity secondary school. In 81% of families, mothers were the primary caregivers (i.e. with whom adolescents spent most of their time), and in 19% of families, fathers were the primary caregivers. Of the 256 adolescents, 176 participated with a parent (*M*_age_ = 46.77, age range = 36–76 years, 90% Dutch), of whom 82% were mothers and 18% were fathers (all were biological parents). Regarding marital status, 75% of participating parents were living together or married, 20% were divorced or separated, and 3% were widowed. Parental educational level varied; 35% completed vocational training, 32% completed a university of applied sciences, and 18% completed university.

### Biweekly measures

#### Adolescent generalized anxiety symptoms

Adolescents and parents completed the Dutch version of the Generalized Anxiety Disorder (GAD) symptoms subscale (Wijsbroek, Hale, Raaijmakers, & Muris, 2005) of the Screen for Child Anxiety Related Emotional Disorders (SCARED; Birmaher et al., 1997). They answered nine items on how the adolescent felt in the past 2 weeks (e.g. “I / My child was worried about how well I/he or she was doing things”) on a scale from 1 (*never*) to 3 (*always*). The reliability of this scale was sufficient to good at the between‐family level (adolescents: *ω* = .87; parents: *ω* = .74) and sufficient at the within‐family level (adolescents: *ω* = .71; parents: *ω* = .70; Geldhof, Preacher, & Zyphur, 2014).

#### Parental intrusiveness

A shortened 3‐item version of the Subscale Intrusiveness of the Level of Expressed Emotion (LEE, Cole & Kazarian, 1993) was used to assess parental intrusiveness. The original subscale consists of 7 items (Hale, Raaijmakers, Gerlsma, & Meeus, 2007), which has been widely used in parenting research (e.g. Hawk, Keijsers, Hale III, & Meeus, 2009). To reduce participant burden in this biweekly study, intrusiveness was measured using three items (instead of seven). The three items with the highest factor loadings from a previous study with Dutch adolescents were selected (Hale et al., 2007). Hence, parents and adolescents rated three items (i.e. “… was involved in everything, interfered with my/child's affairs, and needed to know everything about me/my child”) on a scale from 1 (*never*) to 5 (*very often*). The reliability of this scale was excellent at the between‐family level (adolescents: *ω* = .92; parents: *ω* = .96) and sufficient at the within‐family level (adolescents: *ω* = .74; parents: *ω* = .75).

#### Parental autonomy support

To assess parental autonomy support, parents and adolescents answered a short four‐item scale. Specifically, three items came from the Autonomy Support Scale of the Perceptions of Parents Scale (POPS; Grolnick, Ryan, & Deci, 1991) and one item from Silk et al. (2003) (see Soenens et al., 2007). The four (adolescent‐reported) items were: “… let me make my own plans for things I wanted to do,” “whenever possible, allowed me to choose what to do,” “allowed me to decide things for myself,” and “allowed to choose my own direction in life.*”* Similar items have been used in other intensive longitudinal studies (e.g. Van Der Kaap‐Deeder, Vansteenkiste, Soenens, & Mabbe, 2017). The response scale ranged from 1 (*not at all*) to 5 (*a lot*). For the adolescent‐reported data, the reliability of this scale was adequate at the within‐family level (*ω* = .74) and excellent at the between‐family level (*ω* = .90). For the parent‐reported data, the reliability was excellent at both the within‐family (*ω* = .88) and between‐family level (*ω* = .99).

### Strategy of analysis

#### Compliance

On average, 17.7 (68%) of the 26 biweekly measurements per adolescent (median = 23, 66% of adolescents completed all 26 measurements) were available (for more details, see Boele et al., 2024). Parents completed, on average, 22 (85%) of the 26 weekly measurements (median = 26, 63% of parents completed all 26 measurements). All available data were included because missing data patterns of the data, including scale scores, did not deviate meaningfully from a completely at random pattern. Specifically, the *χ* ^2^/df ratio of Little's MCAR test (Little, 1988) of data in long form with scale scores only was below 3 (adolescent data (*χ* ^2^(52) = 123.421), parent data (*χ* ^2^(11) = 27.417)).

#### Models

**Figure 1:**
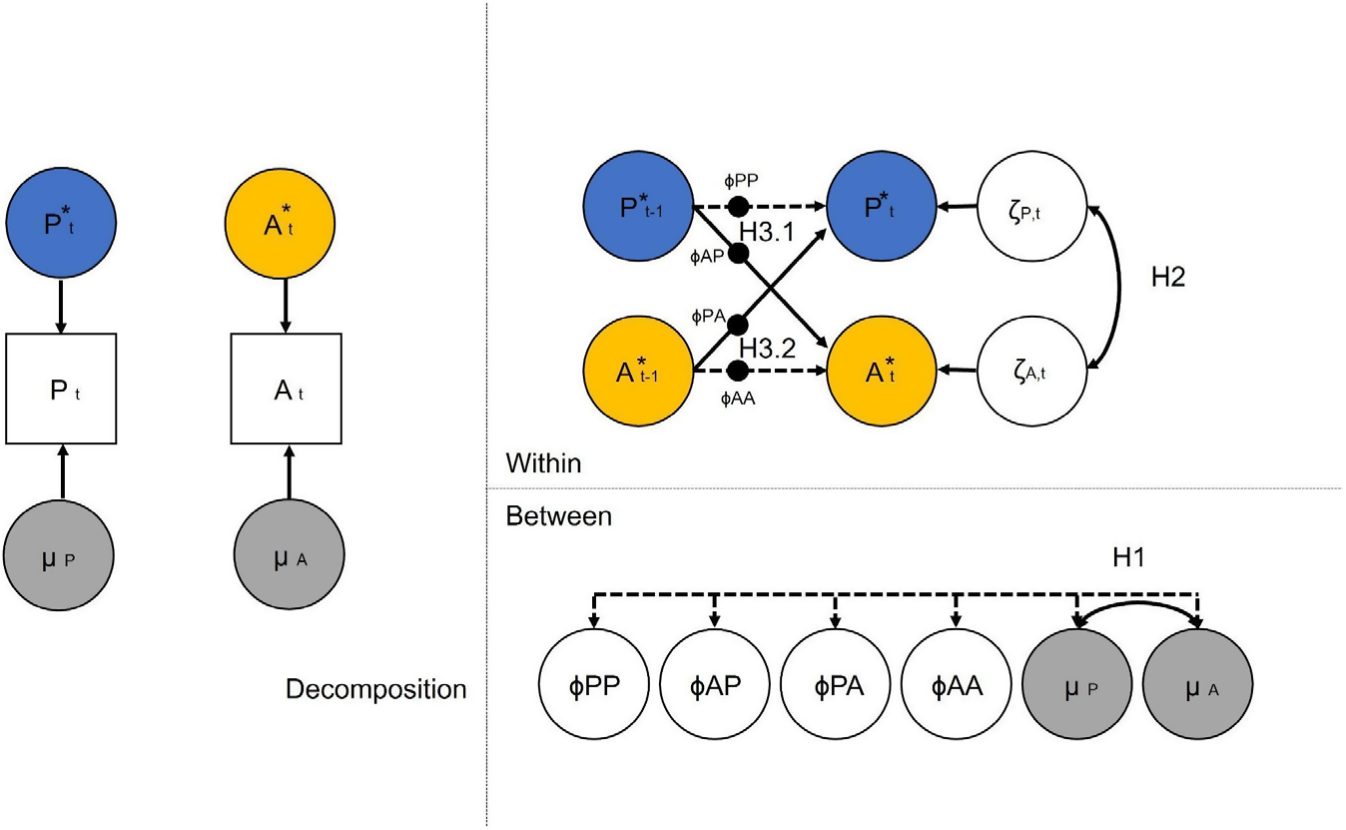
Example of model. A = Adolescent Generalized Anxiety (yellow), P = Parenting (blue). The left panel shows person‐mean centering. Blue and yellow latent factors show within‐family variance in parenting and adolescent anxiety, respectively. Gray latent factors refer to between‐family variance. The right lower panel shows the between‐family correlation (H1). The right upper panel shows the estimation of within‐family correlations (H2). Furthermore, it was estimated whether Parenting (P) predicts changes in anxiety over time (A) (ɸAP; H3.1), and whether anxiety predicts changes in parenting over time (ɸPA; H3.2). The model controls for the autoregressive effects of anxiety (ɸAA) and parenting (ɸPP)

Hypotheses were tested using preregistered Dynamic Structural Equation Modeling (DSEM; McNeish & Hamaker, 2020) in Mplus version 8 (see https://osf.io/tfjue/ for codes and settings; Figure 1). We ran four bivariate models: Model A estimated parental intrusiveness and anxiety associations, while Model B estimated associations with autonomy‐supportive parenting. Both models were run for parent‐ and adolescent‐reports. Due to convergence issues, the models were simplified. For the adolescent‐reported models, no correlations were estimated between the random effects. For the parent‐reported models, the autoregressive effects were estimated as fixed (rather than random) effects.

#### Power analysis

For DSEM, power is derived from the number of individuals and the number of repeated assessments (Schultzberg & Muthén, 2018). This study has 4,537 observations for adolescents (*N* = 256) and 3,892 observations for parents (*N* = 176) in total. In a previous study (Boele et al., 2024), these data were shown to be able to find time‐lagged standardized fixed effects of .05. A standardized time‐lagged effect of .05 is considered a small‐to‐medium effect according to recent guidelines (Orth, Clark et al., 2024), which are typically reported in within‐family parenting studies (for review, see Boele et al., 2020).

## Results

### Descriptive statistics

Descriptive statistics are shown in Table 1. The ICCs indicated that 54%–76% of the variance in the biweekly measures was due to stable between‐family variance (see Table 1), whereas the remaining 24%–46% was due to overtime within‐family fluctuations.

**Table 1:** Descriptive statistics of study variables

Variables	Adolescent‐reported data						Parent‐reported data					
	*M*	*SD*	Range	ICC	*N*	*T*_total_	*M*	*SD*	Range	ICC	*N*	*T*_total_
Adolescent generalized anxiety symptoms	1.60	.53	1.0–3.0	.76	256	4,612	1.40	.38	1.0–3.0	.67	176	3,892
Parental intrusiveness	2.16	.81	1.0–5.0	.71	256	4,545	2.23	.82	1.0–5.0	.67	176	3,894
Parental autonomy support	4.27	.61	1.0–5.0	.64	256	4,459	4.23	.57	1.0–5.0	.54	176	3,901

ICC, intraclass correlation; M, mean; *N*, sample size; *SD*, standard deviation; *T*, number of observations.

As shown in Table 2, the only significant difference between the adolescents' and parents' reports was for anxiety symptoms: The adolescents reported more generalized anxiety symptoms than the parents perceived.

**Table 2:** Mean differences in adolescent‐ and parent reports

	Adolescent‐reported mean	Parent‐reported mean	*T*‐test
Adolescent generalized anxiety symptoms	1.61 (*SD* = .48)	1.42 (*SD* = .34)	*t* = 4.89, *df* = 428, *p* < .01
Parental intrusiveness	2.19 (*SD* = .71)	2.25 (*SD* = .69)	*t* = −0.84, *df* = 426, *p* > .05
Parental autonomy support	4.26 (*SD* = .52)	4.22 (*SD* = .43)	*t* = 0.79, *df* = 426, *p* > .05

*df*, degrees of freedom; *p*, *p*‐value; *SD*, standard deviation; *t*, *t*‐test.

Table 3 shows the between‐family and within‐family level correlations, indicating that there are statistically significant correlations between the parenting practices and adolescents' generalized anxiety symptoms in the expected direction. That is, generalized anxiety symptoms in adolescents were negatively correlated with parental autonomy support and positively correlated with parental intrusiveness.

**Table 3:** Within‐ and between‐family correlations

Variables	Correlations					
	Adolescent‐reported data			Parent‐reported data		
	1.	2.	3.	1.	2.	3.
1. Adolescent generalized anxiety symptoms	–	.12*	−.08*	–	.15*	−.12*
2. Parental intrusiveness	.38*	–	−.22*	.24*	–	−.23*
3. Parental autonomy support	−.23*	−.63*	–	−.10	−.58*	–

Correlations above the diagonal line represent within‐family correlations, and below the diagonal line represent between‐family correlations.

* *p* < .01.

### Preregistered models

#### Adolescent anxiety and parental intrusiveness

At the between‐family level, with both adolescent‐reported (*β* = .38) and parent‐reported (*β* = .24) data, we found that higher levels of parental intrusiveness correlated with higher levels of generalized anxiety symptoms, confirming hypothesis H1a. Moreover, hypothesis H2a was supported: A biweekly increase in parental intrusiveness correlated with a simultaneous increase in adolescents' generalized anxiety symptoms *within families*, found in both the adolescent‐reported (*β* = .11) and parent‐reported data (*β* = .13). No biweekly within‐family lagged effects were found from parental intrusiveness to adolescents' generalized anxiety symptoms, rejecting hypothesis H3.1a. However, positive lagged effects were found from generalized anxiety symptoms to intrusiveness, supporting hypothesis H3.2a, in both the adolescent‐ (*β* = .07) and parent‐reported data (*β* = .05). That is, when adolescents experienced more generalized anxiety symptoms than usual, parents were more intrusive 2 weeks later.

#### Adolescent anxiety and parental autonomy support

At the between‐family level, higher levels of parental autonomy support correlated with lower levels of generalized anxiety symptoms in the adolescent‐reported data (*β* = −.21) but not in the parent‐reported data. Hence, hypothesis H1 was only confirmed with the adolescent‐reported data. Moreover, hypothesis H2b was supported: An increase in parental autonomy support correlated with a decrease in generalized anxiety symptoms within families in both the adolescent‐ (*β* = −.07) and parent‐reported (*β* = −.09) data. However, no lagged effects were found from parental autonomy support to adolescents' generalized anxiety symptoms, rejecting hypothesis H3.1b. Instead, a negative lagged effect was found from adolescents' generalized anxiety symptoms to parental autonomy, but only in the parent‐reported data (*β* = −.08) and not in the adolescent‐reported data (partially supporting hypothesis H3.2b). Hence, when parents perceived that their adolescent was experiencing more generalized anxiety symptoms than usual, they exhibited less autonomy‐supportive behavior 2 weeks later. In sum, for both parental intrusiveness and autonomy support, the findings indicate the expected between‐ and within‐family associations with adolescents' generalized anxiety symptoms. However, the biweekly lagged effects indicate adolescent‐driven processes rather than parent‐driven or reciprocal processes.

#### Sensitivity analysis

To more adequately compare findings of adolescent‐reported versus parent‐reported data, we ran the adolescent‐reported models again (as preregistered) with only those adolescents for whom parent‐reported data were available (*n* = 176). Results were broadly similar to the main findings, except that no significant between‐family correlation was found for parental autonomy support and no significant within‐family lagged effect was found for adolescent generalized anxiety on parental intrusiveness, perhaps due to less statistical power. Descriptive statistics (Table 1), within‐ and between‐family level correlations (Table 2), and DSEM models (Table 3) are available in this study's Tables S1 and S2: https://osf.io/tfjue/.

### Exploratory analyses

To minimize the common method bias (Podsakoff, MacKenzie, Lee, & Podsakoff, 2003), we ran exploratory multi‐informant models. Specifically, adolescent‐ and parent‐reported parenting data were combined in one model. The results are presented in Table S1 and are similar to those in the preregistered main models (see Table 4), with one exception: Parent‐reported intrusiveness did not correlate significantly with adolescent‐reported generalized anxiety symptoms at the between‐family level.

**Table 4:** Dynamic Structural Equation Models (DSEM) with biweekly parenting and adolescent generalized anxiety symptoms

Parameter	Parental intrusiveness						Parental autonomy support					
	Adolescent‐reported data			Parent‐reported data			Adolescent‐reported data			Parent‐reported data		
	Est.	Est. St.	95% CI Est.	Est.	Est. St.	95% CI Est.	Est.	Est. St.	95% CI Est.	Est.	Est. St.	95% CI Est.
Between‐family level												
Correlation (H1)	0.11*	.**38**	**[0.07, 0.16]**	0.05*	.**24**	**[0.02, 0.09]**	−0.04*	**‐ .21**	**[−0.08, −0.01]**	−0.01	−.10	[−0.04, 0.01]
Within‐family level												
Correlated change (H2)	0.01*	.**11**	**[0.01, 0.01]**	0.01*	.**13**	**[0.01, 0.02]**	−0.01*	**‐ .07**	**[−0.01, −0.00]**	−0.01*	**‐ .09**	**[−0.01, −0.00]**
Parenting → Anxiety (H3.1)	0.01	.01	[−0.01, 0.03]	0.02	.03	[−0.00, 0.03]	−0.00	‐ .01	[−0.03, 0.02]	−0.02	‐ .03	[−0.04, 0.00]
Anxiety → Parenting (H3.2)	0.11*	.**07**	**[0.05, 0.17]**	0.11*	.**05**	**[0.02, 0.20]**	−0.04	‐ .03	[−0.09, 0.01]	−0.14*	**−.08**	**[−0.21, − 0.07]**
Parenting → Parenting	0.37*	.**37**	**[0.32, 0.42]**	0.28*	.**28**	**[0.25, 0.32]**	0.34*	.**34**	**[0.28, 0.39]**	0.27*	.**27**	**[0.34, 0.41]**
Anxiety → Anxiety	0.52*	.**52**	**[0.47, 0.57]**	0.38*	.**38**	**[0.34, 0.41]**	0.53*	.**52**	**[0.47, 0.58]**	0.38*	.**38**	**[0.24, 0.31]**

Variances	Est.	*SD*/Est	Est.	*SD*/Est	Est.	*SD*/Est	Est.	*SD*/Est
Parenting → Anxiety	.**00**	**5.59**	.**084**	**19.32**	.**00**	**15.81**	.**01**	**3.54**
Anxiety → Parenting	.**02**	**1.39**	.**002**	**0.40**	.**03**	**4.33**	.**04**	**1.35**

Upper panel Est = unstandardized estimate. Est. St. = standardized estimate (i.e. STDYX standardization). 95% CI = Bayesian credible intervals. Unstandardized estimates with a 95% credible interval that do not contain zero are marked with an asterisk, indicating a meaningful effect. In bold, meaningful effects (i.e. 95% CI not containing zero) in line with our preregistered hypotheses. Lower panel. SD/Est. = standard deviation fixed effect ratio, to inspect whether the variance is meaningful, with a criterion of ≥0.25 (Bolger et al., 2019).

Additionally, we examined how parental autonomy support and intrusiveness are uniquely linked to adolescents' generalized anxiety symptoms because the parenting practices correlate (see Table 3). Specifically, models were estimated that included both parental autonomy support and intrusiveness. These results are displayed in Table S2, which are similar to the preregistered main models (see Table 4). Again, biweekly effects were found from increased generalized anxiety symptoms to less (parent‐reported) autonomy‐supportive parenting (*β* = −.11) and more (adolescent and parent‐reported) intrusive parenting (*β*s = .06). Using parent‐reported data, the effect of generalized anxiety symptoms on autonomy‐supportive parenting was stronger than the effect on intrusive parenting according to the analysis of MCMC chains, which revealed a significant difference between the coefficients, as indicated by a 95% credible interval that did not include zero.

## Discussion

Adolescent anxiety is increasing globally (WHO, 2022). As parenting is a modifiable factor, interventions targeting parenting practices can potentially prevent adolescent anxiety (Yap et al., 2019). However, there is limited evidence on how parenting and adolescent anxiety impact each other over time within families (Boele et al., 2020). Therefore, this study examined the bidirectional links between parenting practices and adolescents' generalized anxiety symptoms using parent‐ and adolescent‐reported data collected biweekly over a year. This meso‐longitudinal design enabled us to study whether more autonomy‐supportive and less intrusive parenting reduces symptoms of generalized anxiety, and whether heightened symptoms of generalized anxiety are followed by more intrusive and less autonomy‐supportive parenting within families.

### Between‐family differences and within‐family dynamics

This study, in which families were followed for a full year, supported most existing research (e.g. Yap et al., 2014). That is, our between‐family findings support the overarching idea that parenting is a significant risk factor for anxiety. Adolescents with parents who were less intrusive and more autonomy‐supportive reported lower levels of generalized anxiety symptoms on average. However, these group‐level estimates do not imply causality at the within‐family level (Hamaker, 2012; Keijsers, 2016).

Inspired by methodological work (Hamaker, Kuipers, & Grasman, 2015; Keijsers, 2016), we conducted within‐family analyses. We found the expected concurrent processes within families between increased parental intrusiveness and heightened generalized anxiety symptoms, as well as between decreased parental autonomy support and heightened generalized anxiety symptoms. Nevertheless, little evidence was found for the time‐lagged parent‐driven effects: when adolescents experienced or parents expressed more autonomy‐supportive or less intrusive behaviors than typical, adolescents generally did not experience more (or fewer) generalized anxiety symptoms 2 weeks later. Recent research has suggested that such parenting processes may operate on smaller timescales, with day‐to‐day effects from more autonomy‐supportive parenting to less negative affect (Bülow et al., 2022) and more psychologically controlling parenting to more emotional problems (Xu & Zheng, 2022). However, the present study found the hypothesized adolescent‐driven effects: when adolescents experienced more generalized anxiety symptoms than typical, parents behaved to be more intrusive and less autonomy‐supportive 2 weeks later. Similar adolescent‐driven effects on parenting were found in a similar study on social anxiety symptoms (Nelemans et al., 2020). As both anxiety and depressive symptoms are characterized by negative affect and negative thinking (Dozois et al., 2009), and anxiety may act as a precursor to depression (Garber & Weersing, 2010), similar effects of increased depressive symptoms on parenting might be expected, which warrant further attention in future research.

The current study is among the first to assess parenting as a bidirectional process using a within‐family design (Boele et al., 2020). Our findings indicate that changes in adolescents' generalized anxiety symptoms trigger changes in their parents' behavior, as reported by both adolescents and parents themselves. Specifically, the adolescent‐driven effects align with interpersonal views on internalizing symptoms (Joiner & Coyne, 1999), positing that internalizing symptoms elicit less supportive behavior from key attachment figures. Moreover, the found effects are in line with the idea that a child's malfunctioning might be a stressor to the parent, limiting their ability to respond in an autonomy‐supportive manner (Belsky, 1984; Van Der Kaap‐Deeder et al., 2019). Another possibility is that parents increase monitoring to shield their child from heightened anxiety but do so in an intrusive manner (Luijk et al. 2023; Segrin et al., 2013). However, the biweekly effect of adolescents' generalized anxiety symptoms was stronger on autonomy‐supportive parenting than on intrusive parenting, suggesting that adolescent anxiety may particularly influence the extent to which parents promote independence rather than being too controlling. Future research may want to further examine the underlying mechanism of why and which parents respond less autonomy‐supportive and more intrusive to increased adolescent anxiety.

### Limitations and future research

Several limitations should be mentioned. First, the findings refer to average sample effects, which may not apply to every family (e.g. Bolger, Zee, Rossignac‐Milon, & Hassin, 2019). Indeed, the present study also found meaningful variance around the average effects. Hence, future studies should examine how the processes between parenting and adolescents' symptoms of generalized anxiety within families are similar and different across families. For example, our findings mainly reflect maternal parenting (81% mothers in the sample), and different findings might emerge for fathers. Fathers are thought to play a particularly significant role in fostering autonomy support (Paquette, 2004), although a meta‐analysis has shown that maternal, but not paternal, overprotection is associated with higher levels of adolescent anxiety (Manuele, Yap, Lin, Pozzi, & Whittle, 2023). Moreover, our nonclinical sample consisted of a relatively homogeneous group of adolescents who were predominantly female and attended a preuniversity secondary school track. Future research could investigate whether these processes differ in more diverse samples (Boele et al., 2024; Boele, Bülow, et al., 2023), including adolescents with generalized anxiety disorders, for instance. Similar adolescent‐driven processes may arise in clinical samples, as clinical levels of anxiety can strain the parent–child relationship (e.g. Joiner & Coyne, 1999). However, regarding the parent‐driven process, parenting could contribute to maintaining clinical anxiety levels (Wood et al., 2003).

Second, parenting processes unfold in daily life on longer timescales (Keijsers & van Roekel, 2019). By detecting biweekly effects, we have most likely tapped into patterns referred to as the “proximal processes” of development (Bronfenbrenner, 2005). Whether short‐ and long‐term influences between parents and adolescents are similar across timescales remains to be investigated. Such a comparison would also allow us to understand *when* parenting practices are related to changes in anxiety, as well as the peculiar situation in which short‐ and long‐term effects may sometimes be counteracting (e.g. de Ruiter, van der Gaag, Jeronimus, & Kunnen, 2019).

Third, we studied adolescent and parent reports of parenting and adolescents' generalized anxiety symptoms. Studies have shown that parents and adolescents differ in their views on parenting and adolescent well‐being (e.g. Janssen, Verkuil, van Houtum, Wever, & Elzinga, 2021; Xu & Zheng, 2022) and discrepant views on parenting might relate to emotional problems (Jager, Yuen, Bornstein, Putnick, & Hendricks, 2014). Future studies could explore how (dis)similarities in these perspectives influence adolescent well‐being. Additionally, observational methods could address the limitations of self‐reports, such as divergent perceptions, social desirability biases (Morsbach & Prinz, 2006), and biased parenting reports when adolescents experience anxiety (Wood et al., 2003).

### Practical implications

The findings of this study have several tentative practical implications. More intrusive and less autonomy‐supportive parenting seems to go hand‐in‐hand with more symptoms of generalized anxiety in adolescents; over time, this process seems particularly adolescent‐driven. Although further evidence is required, including research on the extent to which these adolescent‐driven effects generalize to overlapping emotional problems, such as depressive symptoms, these effects are of importance. In terms of prevention, our findings highlight the need to educate parents on how they might react in less autonomy‐supportive and more intrusive ways to their adolescent's emotional problems. Raising awareness can help parents adopt more need‐supportive responses, preventing support erosion (Joiner & Coyne, 1999). Moreover, the findings highlight the negative impact of adolescent anxiety on the family system, potentially contributing to parental emotional difficulties (Belsky, 1984). Recognizing the impact of adolescent anxiety on parental functioning underscores the need to involve parents in intervention and treatment efforts to prevent further parental malfunctioning. However, for future research, it is important to address the underlying mechanisms and potential variability in these processes across families to refine and personalize intervention strategies.

## Conclusion

Parenting has been proposed as an important modifiable target for reducing anxiety symptoms in adolescents. Although adolescent anxiety is on the rise globally, very few studies have examined the overtime processes linking parenting practices and adolescent generalized anxiety symptoms within families. Our study detected adolescent‐driven effects on a biweekly timescale, such that more generalized anxiety symptoms in adolescents predicted more parental intrusiveness and less autonomy support 2 weeks later. Hence, adolescent anxiety may trigger changes in parental behavior.

## Ethical considerations

The study was approved by the ethical committee of the Faculty of Social and Behavioral Sciences of Tilburg University (Nr. EC‐2019.65t). Active informed consent was obtained from all participants.

**Key points**

- Parenting practices have been recognized as potential risk and protective factors for adolescents' anxiety symptoms.
- In the present study, a novel meso‐longitudinal design was used to investigate how intrusive and autonomy‐supportive parenting are linked to adolescents' generalized anxiety symptoms over time within families.
- The time‐lagged associations suggest adolescent‐driven rather than parent‐driven processes. When adolescents experienced more generalized anxiety symptoms than usual, their parents were less autonomy supportive and more intrusive 2 weeks later.
- These findings highlight the negative impact of adolescent mental health problems on parental functioning, emphasizing the need to support not only adolescents' mental well‐being but also to foster positive and adaptive parent–child dynamics.

## Supporting information


Data S1

## Data Availability

Biweekly data with 26 measurement points per participant may allow identification. To protect the privacy of the participants, data are available from the corresponding author upon reasonable request. Scripts and output are shared on OSF https://osf.io/tfjue.
