# Supplementary material for: Parenting and adolescent anxiety within families: a biweekly longitudinal study
Source: J Child Psychol Psychiatry. 2025 Mar 19;66(9):1414–24. doi: 10.1111/jcpp.14161 (PMC12350824; doi:10.1111/jcpp.14161)
Supplement: Supplementary file 1 — Data S1 [file JCPP-66-1414-s001.docx]

**Supporting Information**

**Table S1**

*Exploratory DSEM Analyses with Multi-Informant Parenting Data and Adolescent-Reported Generalized Anxiety Symptoms (n = 176)*

|  | **Parental Intrusiveness** | | | **Parental Autonomy Support** | | |
| --- | --- | --- | --- | --- | --- | --- |
| **Parameter** | **Est.** | **Est. St.** | **95% CI Est.** | **Est.** | **Est. St.** | **95% CI Est.** |
| ***Between-family level*** |  |  |  |  |  |  |
| Correlation parenting (AR) and anxiety  (AR) | 0.08* | .26 | [0.02, 0.14] | -0.04* | - .21 | [-0.09, -0.00] |
| Correlation parenting (PR) and anxiety  (AR) | -0.00 | - .01 | [-0.06, 0.05] | 0.00 | .02 | [-0.04, 0.11] |
| ***Within-family level*** |  |  |  |  |  |  |
| Correlated change parenting (AR) and anxiety (AR) | 0.01* | .11 | [0.01, 0.01] | -0.01* | - .07 | [-0.01, -0.00] |
| Correlated change parenting (PR) and anxiety (AR) | 0.01* | .05 | [0.00, 0.01] | -0.01* | - .08 | [-0.01, -0.00] |
| Parenting (AR) → Anxiety (AR) | -0.01 | - .02 | [-0.03, 0.01] | 0.01 | .02 | [-0.01, 0.04] |
| Parenting (PR) → Anxiety (AR) | 0.02 | .03 | [-0.00, 0.04] | -0 .01 | - .02 | [-0.04, 0.01] |
| Anxiety (AR) → Parenting (AR) | 0.09* | .06 | [0.03, 0.15] | -0.03 | - .02 | [-0.08, 0.02] |
| Anxiety (AR) → Parenting (PR) | 0.10* | .07 | [0.04, 0.17] | -0.10* | - .08 | [-0.15, -0.05] |
| Parenting (AR) → Parenting (AR) | 0.38* | .38 | [0.31, 0.44] | 0.35* | .35 | [0.39, 0.42] |
| Parenting (PR) → Parenting (PR) | 0.28* | .28 | [0.23, 0.34] | 0.26* | .26 | [0.21, 0.32] |
| Anxiety (AR) → Anxiety (AR) | 0.52* | .52 | [0.46, 0.59] | 0.52* | .51 | [0.46, 0.58] |

*Note*. AR = adolescent-reported data. PR = parent-reported data. Est = unstandardized estimate. Est. St. = standardized estimate (i.e., STDYX standardization). 95% CI = Bayesian Credible Intervals of the unstandardized estimate. Unstandardized estimates with a 95% credible interval that does not contain zero are marked with an asterisk, indicating a meaningful effect. Adolescent- and parent-reported intrusiveness correlated at the within-family (*r* = .08, 95% CI [.01 - .02]) and between-family level (*r* = .27, 95% CI [.05 - .22]. Adolescent- and parent-reported autonomy support also correlated at the within-family (*r* = .07, 95% CI [.00 - .01]) and between-family level (r = .38, 95% CI [.04 - .11]).

**Table S2**

*Exploratory DSEM Analyses Including Both Parenting Practices*

|  | **Adolescent-reported data** | | | **Parent-reported data** | | |
| --- | --- | --- | --- | --- | --- | --- |
| **Parameter** | **Est.** | **Est. St.** | **95% CI Est.** | **Est.** | **Est. St.** | **95% CI Est.** |
| ***Between-family level*** |  |  |  |  |  |  |
| Correlation autonomy support and anxiety | -0.05* | -.23 | [-0.09, -0.01] | -0.02 | -.15 | [-0.04, 0.00] |
| Correlation intrusiveness and anxiety | 0.08* | .27 | [0.03, 0.14] | 0.05* | .25 | [0.02, 0.09] |
| Correlation autonomy support intrusiveness | -0.22* | -.65 | [-0.30, -0.16] | -0.17* | -.62 | [-0.23, -0.12] |
| ***Within-family level*** |  |  |  |  |  |  |
| Correlated change autonomy support and anxiety | -0.01* | -.07 | [-0.01, -0.00] | -0.01* | -.09 | [-0.01, -0.00] |
| Correlated change intrusiveness and anxiety | 0.01* | .11 | [0.01, 0.01] | 0.01* | .14 | [0.01, 0.02] |
| Correlated change autonomy support and intrusiveness | -0.02* | -.17 | [-0.03, -0.02] | -0.03* | -.19 | [-0.04, -0.03] |
| Autonomy support → Anxiety | 0.01 | .02 | [-0.01, 0.04] | -0.01 | -.01 | [-0.03, 0.01] |
| Intrusiveness → Anxiety | -0.01 | -.02 | [-0.04, 0.01] | 0.01 | .01 | [-0.01, 0.03] |
| Anxiety → Autonomy support | -0.03 | -.02 | [-0.07, 0.02] | -0.17* | -.11 | [-0.24, -0.11] |
| Anxiety → Intrusiveness | 0.09* | .06 | [0.03, 0.15] | 0.11* | .06 | [0.03, 0.18] |
| Autonomy support → Autonomy support | 0.34* | .34 | [0.28, 0.40] | 0.25* | .25 | [0.19, 0.30] |
| Intrusiveness → Intrusiveness | 0.36* | .36 | [0.30, 0.42] | 0.27* | .27 | [0.21, 0.32] |
| Anxiety → Anxiety | 0.53* | .52 | [0.47, 0.59] | 0.39* | .38 | [0.33, 0.45] |

*Note*. Est = unstandardized estimate. Est. St. = standardized estimate (i.e., STDYX standardization). 95% CI = Bayesian Credible Intervals of the unstandardized estimate. Unstandardized estimates with a 95% credible interval that does not contain zero are marked with an asterisk, indicating a meaningful effect.
